# Low-polarity untargeted metabolomic profiling as a tool to gain insight into seminal fluid

**DOI:** 10.1007/s11306-023-02020-y

**Published:** 2023-06-05

**Authors:** Eulalia Olesti, Julien Boccard, Rita Rahban, Sergey Girel, Natalia E. Moskaleva, Fanny Zufferey, Michel F. Rossier, Serge Nef, Serge Rudaz, Víctor González-Ruiz

**Affiliations:** 1 School of Pharmaceutical Sciences, University of Geneva, Geneva, Switzerland; 2 Institute of Pharmaceutical Sciences of Western Switzerland, University of Geneva, Geneva, Switzerland; 3 Swiss Centre for Applied Human Toxicology (SCAHT), Basel, Switzerland; 4 Department of Genetic Medicine and Development, Faculty of Medicine, University of Geneva, Geneva, Switzerland; 5 Laboratory of Pharmacokinetics and Metabolomic Analysis, Institute of Translational Medicine and Biotechnology, I. M. Sechenov First Moscow State Medical University, Moscow, Russian Federation; 6 Service of Clinical Chemistry & Toxicology, Central Institute of Hospitals, Hospital of Valais, Sion, Switzerland; 7 Department of Internal Medicine, Faculty of Medicine, University of Geneva, Geneva, Switzerland; 8 Present Address: Centro de Metabolómica y Bioanálisis (CEMBIO), Facultad de Farmacia, Universidad San Pablo-CEU, CEU Universities, Madrid, Spain

**Keywords:** Semen quality, Seminal fluid, Sperm, Metabolomics

## Abstract

**Introduction:**

A decrease in sperm cell count has been observed along the last several decades, especially in the most developed regions of the world. The use of metabolomics to study the composition of the seminal fluid is a promising approach to gain access to the molecular mechanisms underlying this fact.

**Objectives:**

In the present work, we aimed at relating metabolomic profiles of young healthy men to their semen quality parameters obtained from conventional microscopic analysis.

**Methods:**

An untargeted metabolomics approach focusing on low- to mid-polarity compounds was used to analyze a subset of seminal fluid samples from a cohort of over 2700 young healthy men.

**Results:**

Our results show that a broad metabolic profiling comprising several families of compounds (including acyl-carnitines, steroids, and other lipids) can contribute to effectively distinguish samples provided by individuals exhibiting low or high absolute sperm counts.

**Conclusion:**

A number of metabolites involved in sexual development and function, signaling, and energy metabolism were highlighted as being distinctive of samples coming from either group, proving untargeted metabolomics as a promising tool to better understand the pathophysiological processes responsible for male fertility impairment.

**Supplementary Information:**

The online version contains supplementary material available at 10.1007/s11306-023-02020-y.

## Introduction

Over two thirds of people in the world live in regions where the Total Fertility Rate (TFR, defined by the number of live births per woman) have been constantly declining over the last decades. In most European countries, the US and Japan, the TFR is now below the replacement level needed to reliably sustain populations (Skakkebæk et al., 2022; World Population Prospects, 2022), making an increasing number of couples need to turn to assisted reproduction techniques. Hence, infertility (the failure to establish a pregnancy after 12 months of regular, unprotected sexual intercourse (Zegers-Hochschild et al., 2017)) and its future trends are becoming an increasingly worrying issue worldwide (Vollset et al., 2020). Besides diverse socioeconomical factors (*e.g.* delays in couples’ pregnancy planning) there is a strong body of evidence supporting that one of the major reasons for such a health issue is impaired semen quality (Levine et al., 2017). Although such a statement has been the subject of some debate (Boulicault et al., 2022; Jørgensen et al., 2021), the most recent studies have evidenced a global and constant decrease of sperm count during the past decades (Levine et al., 2022).

From the biological point of view, the seminal fluid carries the sperm cells (or spermatozoa) in the seminal plasma –the mix of secretions coming from seminal vesicles, prostate, and bulbourethral and periurethral glands (Drabovich et al., 2014). In humans, spermatozoa only account for ~ 5% of the total volume of the ejaculate and, being small cells with a minute amount of cytoplasm and the ability to move, they rely on the seminal plasma to be nourished, protected from the environment, and to fertilize the oocyte (Robertson & Sharkey, 2016; Schjenken & Robertson, 2020; Turunen et al., 2022). To fulfill these roles, the seminal plasma contains a large variety of nutrients, buffering ions, immunomodulators, and signaling molecules (Poiani, 2006). Along with its intrinsic high viscosity and protein content (Owen & Katz, 2005) it renders the seminal liquid a challenging matrix as a subject for bioanalyses.

Despite its analytical complexity, its close functional and anatomical relationship with the male reproductive organs makes semen an appealing biofluid to develop diagnostic approaches and to study the etiology and the mechanisms underlying alterations of the male genital tract (Blaurock et al., 2022). Although this type of applications has been developed in the proteomics field (Camargo et al., 2018; Drabovich et al., 2014; Druart & Graaf, 2018; Martins et al., 2020; Milardi et al., 2013; Samanta et al., 2018), the body of studies dedicated to disentangling the metabolome composition of human seminal fluid and its correlation with semen quality is quite scarce. There is a remarkable number of publications focusing on the targeted analysis of up to ten steroid hormones (Balladová, 2015; Hampl et al., 2013; Vitku et al., 2015, 2017; Ying et al., 1983; Zalata et al., 2014), but research devoted to an untargeted (> 50) determination of steroid hormones in seminal liquid remains limited (Olesti et al., 2020). The number of studies increasing the chemical coverage from a fully untargeted perspective, is even more restricted (Blaurock et al., 2022; Buszewska-Forajta et al., 2022; Engel et al., 2019; Mehrparavar et al., 2019; Qiao et al., 2017; Serri et al., 2022; Xu et al., 2020; Zeng et al., 2018). It becomes thus evident that there is a lack of knowledge about small molecule-composition of human seminal fluid.

In this context, a dedicated approach was previously developed to determine and annotate up to nearly 200 steroids in seminal fluid (Olesti et al., 2020, 2021). This methodology exploits an optimized SPE-based sample preparation, followed by RP-HRMS analysis of the samples. Highly reliable level 2 or level 1 annotations (Schymanski et al., 2014) are then made possible by DynaSti, a retention time database containing 92 experimentally determined retention times (RT) and over 100 in silico calculated ones (Codesido et al., 2019). Despite the biological relevance of steroids in the seminal fluid, the complexity of this matrix goes far beyond what can be monitored through a single family of compounds. Additional mediators such as oxylipins are also present and account for an adequate sperm functionality. A number of other molecules with different degrees of lipophilicity are present in the seminal fluid and play an important role during spermatogenesis (sperm production), during their maturation throughout the epididymis, or even on the response of the female epithelium (Poiani, 2006; Robertson, 2005). Tracking such molecules in the search for differences between low- and high-quality semen samples using an untargeted approach has the advantage of providing a more complete picture of potential changes in their chemical composition. Moreover, it becomes particularly useful in the context of exploratory studies because no previous knowledge about the concerned metabolic pathways is needed.

For this purpose, a strategy aiming to broaden and complement the metabolic coverage provided by the existing LC–MS method covering 200 steroids has been developed and implemented thanks to the use of several different *in-house* analyzed libraries of compounds. Such libraries contained over 800 reference standards for which accurate masses, RTs, and fragmentation patterns have been experimentally measured, enabling the reliable identification of the metabolites present in seminal fluid at level 1 of confidence (Blaženović et al., 2018; M.I.T. Group, 2020; Sumner et al., 2007). This annotation effort has allowed to distinguish a wide variety of metabolites accounting for the difference between low- and high-sperm count individuals in an epidemiological study conducted on young Swiss men.

## Materials and methods

### Study population and semen analysis

From September 2005 to June 2017, a nationwide cross-sectional study was conducted on 2731 men aged between 18 and 22 years coming from all regions of Switzerland. Volunteers were recruited upon their participation in a mandatory short military camp before their potential enrollment in the Swiss military service. The details about the study can be found elsewhere (Rahban et al., 2019). Volunteers provided urine, blood plasma and a semen sample and filled a comprehensive questionnaire about their general and reproductive health as well as their lifestyle habits. Upon collection, semen samples were incubated at 37 °C for 20–40 min to allow liquefaction as recommended in the WHO manual for semen analysis (World Health Organization, 2010). Aliquots (5 µL) of the semen sample were then transferred into a 20 µm-deep counting chamber (Leja Products, The Netherlands) and analyzed using a Computer Assisted Sperm Analyzer (CASA, Sperm Class Analyzer- SCA, Microptic, Spain). Semen parameters such as concentration (million/mL), Total Sperm Count (TSC – million/ejaculate), and sperm motility (%) were recorded. Classes of sperm motility were determined according to previously described kinematic parameters (Mortimer, 1997) and divided into the following groups: progressive (rapid and slow, type a and b, respectively), non-progressive (type c) and static (type d). Fixed and Papanicolaou stained smears were prepared for sperm morphology assessment, either using the CASA or by a single trained technician according to the stricter criteria (Menkveld et al., 1990). The semen samples were then centrifuged at 700 × g for 10 min, and the supernatant, representing the seminal fluid, was collected. Aliquots of blood serum, urine and seminal fluid were stored at − 80 °C until use. The study was approved by the ethical committees of the cantons of Vaud (17–01-2005, 01/02), Zürich (EK-StV-Nr. 27–2006), Ticino (Rif.CE 1886) and Geneva (2016–01674) in Switzerland.

### Chemicals and reagents

Phosphoric acid (H_3_PO_4_) analytical grade was purchased from Sigma-Aldrich (Buchs, Switzerland). Formic acid (FA) was obtained from Biosolve (Valkenswaard, The Netherlands), and acetonitrile (MeCN), water (H_2_O), and methanol (MeOH) from Fisher Scientific (Loughborough, UK). All the solvents and additives were UPLC-MS grade.

### Sample preparation

Seminal fluids were extracted as previously published (Olesti et al., 2020). Briefly, 200 µL of seminal fluid were acidified with 500 μL of aqueous 4% H_3_PO_4_ and then loaded onto SPE HLB μElution plates (Waters, Milford, MA, USA) using a positive pressure manifold (PRESSURE + 96, Biotage AB, Uppsala, Sweden). Samples were washed with 400 μL of H_2_O:MeOH (95:5) and then eluted with 50 μL of H_2_O:MeCN (10:90). Eluates were evaporated to dryness (SpeedVac, Thermo Fischer Scientific, Waltham, MA, USA) and reconstituted in 100 μL of H_2_O:MeOH (50:50).

### Sequence

The analytical sequence was split into two separate batches comprising 140 and 142 samples, analysed consecutively after MS cleaning. Each one of the batches contained blanks, system suitability tests, conditioning and long-term QC samples (Pezzatti et al., 2020). QC/dQC pairs were analysed every 8 study samples. Long-term QCs were extracted as detailed in Sect. 2.2 from a pool of healthy donors and routinely used to check analytical quality. Intra-study QC samples were prepared by pooling aliquots of all the extracts from the study samples and also used as conditioning samples. Diluted QC samples were made by diluting QC samples with injection solvent by a 1:1 ratio.

### LC–MS analyses

Chromatography was performed on a Waters H-Class Acquity UPLC system composed of a quaternary pump, a column manager and an FTN autosampler (Waters Corporation, Milford, MA, USA). Samples were separated on a Kinetex C18 column (150 × 2.1 mm, 1.7 µm) and the corresponding SecurityGuard Ultra precolumn and holder (Phenomenex, Torrance, USA). Solvent A was H_2_O and solvent B was MeCN, both containing 0.1% formic acid. The column temperature and flow rate were set at 30 ºC and 300 µL min^–1^, respectively. The gradient elution was as follows: 2 to 100% B in 14 min, hold for 3 min, then back to 2% B in 0.1 min and re-equilibration of the column for 7.9 min. The UPLC system was coupled to a maXis 3G Q-TOF high-resolution mass spectrometer from Bruker (Bruker Daltonik GmbH, Bremen, Germany) through an electrospray interface (ESI). The capillary voltage was set at –4.7 kV (ESI +), drying gas temperature was 225 ºC, drying gas flow rate was set at 5.50 L min^–1^ and nebulizing gas pressure was 1.8 bar. Transfer time was set at 40 µs and pre-pulse storage duration at 7.0 µs. Data between 50 and 1000 m/z were acquired in profile mode at a rate of 2 Hz. Formate adducts in the 90–1247 m/z range were employed for in-run automatic calibration using the quadratic plus high-precision calibration algorithm provided by the instrument’s manufacturer. MS and UPLC control and data acquisition were performed through the HyStar v3.2 SR2 software (Bruker Daltonik) running the Waters Acquity UPLC v.1.5 plug-in.

### Data pre-processing and metabolite annotation

Run alignment, peak-picking and annotation were performed on Progenesis QI v2.3 (Nonlinear Dynamics, Waters, Newcastle upon Tyne, UK). dQC/QC ratios were used to filter out analytically unreliable features using *in-house* developed Java scripts. A threshold of 20% was applied as the upper limit of the dQC/dQC ratio relative standard deviation. In addition, a dQC/QC ratio between 0.2 and 0.8 was considered acceptable around the theoretical value of 0.5 (1:1 dilution), and signals outside this range were removed. Intra-batch and long-term QCs were used to correct analytical drift by using scripts developed under the MATLAB® 8 environment (The MathWorks, Natick, USA). LOESS regression involving a linear fit and an initial smoothing span of 0.75 was used for intra- and inter-batch normalization based on QCs. The span value was then optimized using cross-validation. Probabilistic Quotient Normalization (PQN) using median QC values as reference was applied to ensure the comparability of the samples under study (Codesido et al., 2019; Robertson, 2005).

Peaks were identified by matching their RTs, accurate masses and isotopic patterns to a database of standards. Such a database was built in-lab, analyzing a set of libraries of reference compounds under the same conditions as the samples, and comprising: 634 chemical standards (MSMLS, Sigma-Aldrich), 192 steroid standards (Sigma-Aldrich; Steraloids, Newport, USA; and Lipomed Arlesheim, Switzerland), 29 acyl-carnitines (Sigma-Aldrich) and 65 oxylipins (synthetized and kindly provided by Prof. Vladimir Bezuglov, Shemykin-Ovchinikov Institute of Bioorganic Chemistry RAS, Moscow). Six compounds were annotated at level 2 based only on their isotopic profiles and fragmentation patterns retrieved from available databases (MONA and Waters Metabolic Profiling CCS Database, as detailed in Supplementary Information 1 for significant metabolites).

### Data analysis

Unit variance scaling was used as a pre-treatment. Probabilistic Quotient Normalization (PQN) (Dieterle et al., 2006) Principal Component Analysis (PCA), Monte Carlo Uninformative Variable Elimination-Partial Least Squares (MCUVE-PLS) (Han et al., 2008) and Orthogonal Partial Least Squares-Discriminant Analysis (OPLS-DA) models were calculated using combinations of toolboxes and in-house functions in MATLAB® 8. MCUVE-PLS was carried out using the libPLS 1.98 package (Li et al., 2018) using an ensemble of 10^4^ models with a ratio of training samples of 0.7. A threshold of 1.5 was applied to reliability index values to remove variables considered as uninformative.

PLS prediction performance was evaluated using leave-one-out cross-validation to compute the discriminant Q^2^ (DQ^2^) index (Westerhuis et al., 2008). The latter is an adaptation of the standard Q^2^ value to discriminant analysis that does not penalize class predictions beyond the class label value.

## Results and discussion

Semen samples were collected from a national cohort of more than 2700 Swiss young men, recruited during military conscription (Rahban et al., 2019). The primary objective was to obtain a more comprehensive metabolic profile of seminal fluids, in addition to the steroidomic fingerprint, to provide some insights into the metabolic and signaling pathways underlying differences between high and low sperm count volunteers.

### Semen quality evaluation

Since the study cohort is representative of the general population of Swiss young men, semen parameters measured on the 2731 seminal fluids were investigated to get an objective and quantitative evaluation of semen quality (World Health Organization, 2010). Eight semen characteristics considered as the most relevant criteria describing motility, concentration and morphology, were selected (Guzick et al., 2001). PCA was then computed to obtain an overview of the collected samples distribution according to these parameters.

Four principal components (PCs) were considered for interpretation, summarizing 33.5%, 21.0%, 19.2%, 11.3% of the total variance, respectively, for a cumulated variance of 85.0%, as relevant trends could be associated with each of these PCs. PC1 could be related to a trend following motility with a clear contribution of motile spermatozoa, independently of their efficacy, *i.e.* variables associated with the number of sperm with a slow progressive motility (SP), rapid progressive motility (QP), non-progressing (NP), and hyperactive (HY) spermatozoa, as opposed to static sperm (ST).

**Fig. 1:**
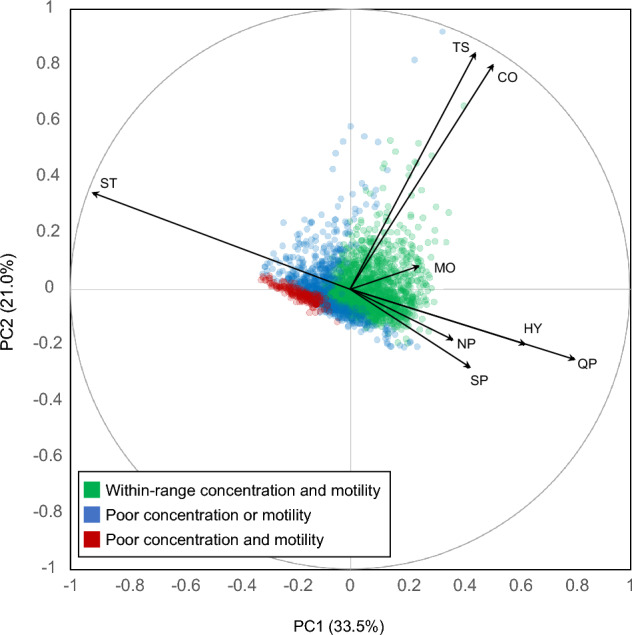
PCA biplot showing the distribution of the 2731 semen samples from the study according to semen parameters. The four first principal components were related to motility, concentration, and morphology. Samples are colour-coded according to reference values established by the WHO for fertile men as detailed in the text. Semen parameters contributions are displayed as arrows: SP slow progressing, QP quick progressing, NP non-progressing, HY hyperactive spermatozoa, CO sperm concentration, TS: total sperm count, MO morphology

PC2 could be linked to differences in overall number of spermatozoa with marked positive contributions of sperm concentration (CO) and total number of spermatozoa (TS) showing a correlated trend between these two descriptors, which therefore seem to be closely related, suggesting a comparable initial volume of seminal fluid during sample collection. The distribution of the samples on the first principal plane (PC1 vs. PC2) is illustrated on Fig. 1, using a color scheme related to semen quality. By examining this biplot, it can be seen how the samples with the lowest sperm concentration and sperm motility (red circles) are clustered in a thin group extending along PC1 and PC2, thus meaning that samples having both sperm motility and concentration below the WHO threshold (40% motile spermatozoa and 15 million cells/mL) are quite homogeneous. On the contrary, samples having only one or none of these two variables under the reference limits, are much more spread over the PCA space.

PC3 could be associated with a second motility pattern separating progressively rapid, slow, non-progressive, static, and hyperactive sperms. Interestingly, PC4 was mainly driven by the morphology parameter (MO). Taken together these four principal components helped to extract major trends in semen parameters that were linked to relevant functionality characteristics in an unsupervised and objective manner.

### Sample selection

**Fig. 2:**
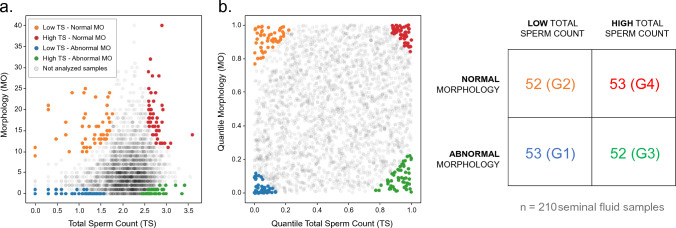
Absolute **a** and quantiles **b** plots showing the distribution of the samples of the cohort under study when stratified according to their morphology (MO) and their total sperm count (TS). Right: total amount of samples in each group

Samples were then stratified according to their position in the quantile distribution for TS and MO within the 2731 samples to maximize potential biochemical composition differences by considering clearly contrasted conditions. Then, the 200 most extreme samples of the cohort according to these two variables were selected to create four distinct groups representing different degrees of semen quality: 50 samples with high TS and normal MO, 50 with low TS and normal MO, 50 with high TS and abnormal MO and 50 with low TS and abnormal MO. (Fig. 2). Such a knowledge would later allow the pathophysiological processes behind different types of sub-fertility to be more efficiently highlighted by finding metabolic features characterising different conditions of good and poor-quality samples. An additional amount of 5% extra samples (10 in total) were added to the 200 originally selected ones to account for losses during sample preparation, and analysis. The resulting 210 samples were submitted to chemical analysis as detailed in the Materials and Methods section.

The analytical platform performed with negligeable retention time and intensity drift along the sequence of each batch and between the two batches. In all, the pre-processing of all the 384 individual runs yielded over 120.000 features. Such features came from 51% of [M + H]^+^ adducts, 24% of [M + Na]^+^, 17% of [M + H-H_2_O]^+^, and 8% of [M + H-2H_2_O]^+^. Features filtering, within- and between-batch drift correction, and sample normalization procedures were carried out as described in the Materials and Methods section. Metabolite annotation was then performed and a total of 210 features could be identified based on experimental accurate mass and retention time match: 110 from the MSMLS library, 69 steroids, 22 oxylipins and 9 acylcarnitines (Supplementary Information Table 1). The annotation effort was limited to these groups of compounds since the sample preparation and the LC were mainly targeting low- to mid-polarity compounds.

Two OPLS-DA models based on the whole set of 210 identified metabolites were then evaluated (Bylesjö et al., 2006) and leave-one-out cross-validation was performed to assess the optimal model size and predictive ability. Because of its ability to separate Y-predictive from Y-orthogonal variations, OPLS-DA extends the interpretability of OPLS (Trygg & Wold, 2002) to discriminant analysis.

A first OPLS-DA model was computed to compare samples characterized by abnormal (G1 & G3) vs. normal (G2 & G4) morphology whatever their sperm counts, as evaluated using CASA (Fig. 2). The model was found optimal with two latent variables (one predictive and one orthogonal) using leave-one-out cross-validation and characterized by R^2^Y = 0.298, DQ^2^Y = 0.015, Accuracy_CV_ = 58.1%. The very low prediction performance of the model revealed that spermatozoa morphology could not be significantly related to any metabolic alterations in the seminal fluid.

A second OPLS-DA model was then evaluated to compare samples characterized by low (G1 & G2) vs. high (G3 & G4) sperm counts whatever their morphology, as evaluated using CASA (Fig. 2). The model was found optimal with three latent variables (one predictive and two orthogonal) using leave-one-out cross-validation and characterized by R^2^Y = 0.603, DQ^2^Y = 0.457, Accuracy_CV_ = 80.0%. This model showed moderate but promising prediction performance despite significant heterogeneity within the groups compared. A strategy to discard non-informative variables was thus carried out in order to remove the less relevant or highly varying signals from the metabolic profiles by relying on the MCUVE-PLS method. The objective of such an approach is to leave aside signals that are difficult to interpret because they exhibit too much variability, and to focus on a subset of robust variables to distinguish the different classes of observations. An ensemble learning strategy based on resampling is implemented to limit the risk of overfitting. This promotes the stability and therefore the interpretability of the resulting multivariate model based on a stable subset of informative variables.

### Uninformative variable elimination

MCUVE-PLS is a variable selection method evaluating both the amplitude and stability of PLS regression coefficients using a Monte Carlo resampling strategy. A reliability index based on these two characteristics is computed for each variable to assess its predictive value estimated from a large ensemble of models generated by randomly selecting training sets from the initial data. This parameter then serves as a selection threshold to leave out variables that are considered uninformative due to low amplitude coefficient and/or too much variability. This method was reported as an efficient variable elimination strategy by offering a robust estimation of the coefficient’s amplitude and variability, thus decreasing the risk of overfitting. This approach also has the advantage of not just focusing on a limited subset of the most predictive variables, but also of retaining all the signals that contribute to distinguish situations of interest such as different experimental conditions. This is particularly relevant in the context of metabolomic analyses because it allows all the potentially modulated metabolites to be kept in the model, thus offering a more complete biological interpretation by integrating the different molecular actors of the involved pathways.

**Fig. 3:**
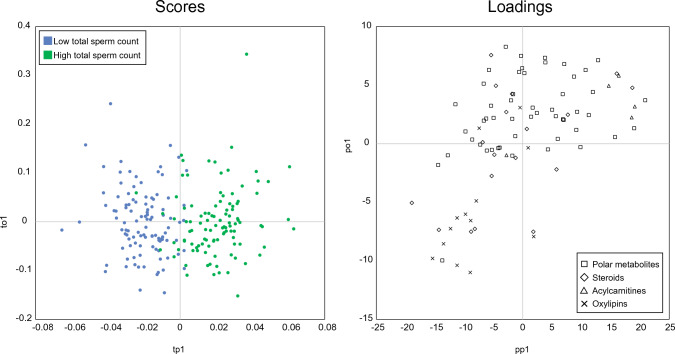
Scores (left) and loadings (right) plots of the OPLS-DA resulting from using the 87 metabolites retained by the MCUVE-PLS strategy when comparing low *vs* high sperm count groups. Symbols in the loadings represent the different annotation libraries

A reliability index cut-off of 1.5 was found suitable to remove uninformative metabolites, while preserving biological information, thus leading to a subset of 87 variables (41.4% of the initial dataset). A refined model (Fig. 3) was then evaluated and found to be robust with three latent variables (one predictive and two orthogonal) based on cross-validation. Moreover, improved prediction accuracy was observed, with R^2^Y = 0.678, DQ^2^Y = 0.610, Accuracy_CV_ = 87.6%. Biological interpretation was then carried out based on loadings associated with variables contributions.

### Biological implications

**Fig. 4:**
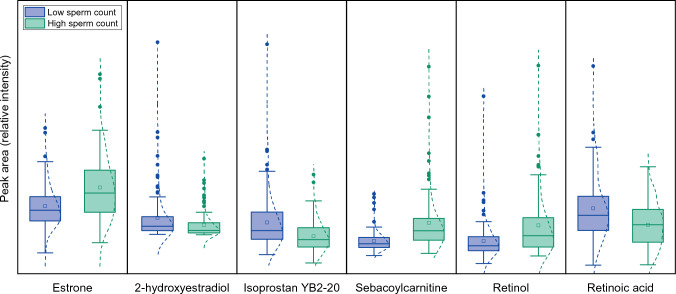
Examples of metabolites highlighted by the multivariate analysis strategy as differences between individuals having low *vs.* high sperm count. Relative intensity scales are in arbitrary peak area units

**Fig. 5:**
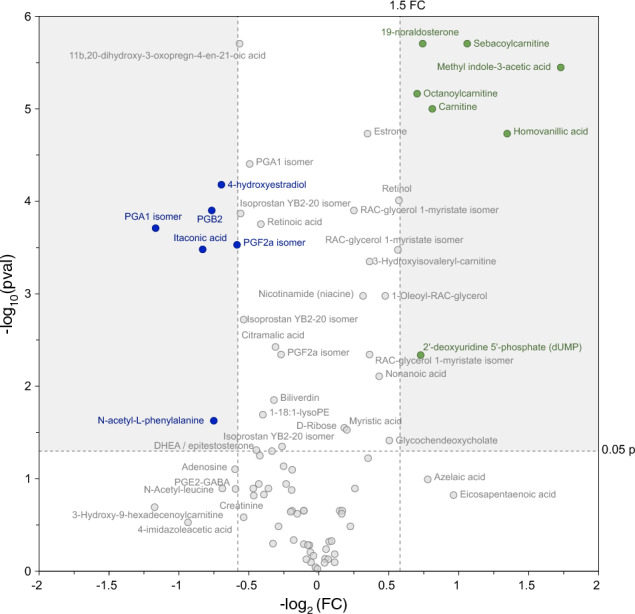
Volcano plot showing the most discriminant metabolites allowing to distinguish seminal plasma samples coming from low or high sperm count donors. Positive FC values correspond to those metabolites showing higher relative concentrations in high sperm count samples

The refined OPLS-DA model and volcano plot allowed to highlight a panel of metabolites whose concentrations were different in samples coming from volunteers with low total sperm count from those with high total sperm count (Figs. 4 and 5).

Steroids that can be found in seminal fluid do not only originate from the local production of androgens by Leydig cells, but also originate from the systemic blood circulation passing through the blood-testis barrier. These molecules are well-known to play a crucial role in spermatogenesis (Hampl et al., 2013) and, thus, they were one of the priority groups of interest. While androgens show a positive correlation with sperm production, the opposite effect has been observed for estrogens (Vitku et al., 2017). In the present study, we found that the levels of 2-hydroxyestradiol, an estradiol metabolite, were higher in the individuals with the lowest sperm count, in good agreement with the previous knowledge (Hampl et al., 2013). Interestingly, the levels of the estradiol precursor and metabolite estrone were found to be larger in individuals with higher sperm count, suggesting a deregulation of the interconversion of estrone and estradiol via the 17β-hydroxysteroid dehydrogenase.

Monitoring of oxylipin-type compounds revealed a high abundance of these molecules in seminal fluid. Indeed, over 200 features could putatively be assigned to prostaglandins based on their formulae and retention times. Nevertheless, we were able to reliably annotate only 22 of these compounds by matching their accurate masses and retention times to our database of standards. Although prostaglandins play a relevant role (Cosentino et al., 1984), it remains unclear how many of them exactly relate to semen quality, with both low and high levels being deleterious for sperm maturation and activity (Isidori et al., 1980). Prostaglandins E, for instance, have been shown to improve sperm motility. On the contrary, higher levels of prostaglandin A1, a pro-inflammatory one, can be related to inflammatory response, increased ROS stress and, thus, a less efficient spermatogenesis. In the case of isoprostans, they are related to sperm immaturity and oxidative damage (Signorini et al., 2020), thus explaining why lower levels can be found in the better semen quality samples.

Carnitines also show remarkable differences between low and high sperm count volunteers. Acylcarnitines transport cytoplasm acyl-groups to the mitochondria to be used in energy production during beta-oxidation. The concentration of carnitines found in the male reproductive tract is unusually high. The transport of carnitine from blood plasma to the epididymis is mediated by specific active transporters in Sertoli cells and seminiferous tubules. Since such increased levels can be found especially in the epididymis, this points to its contribution to the maturation of sperm cells (Mongioi et al., 2016). In this direction, our results show that, indeed, higher carnitine and acylcarnitine levels can be found in the seminal plasma of individuals with higher sperm count. This could be caused by differences in diet carnitine intake, or different transporter activity among the individuals. These results support the role of carnitine and its derivatives in improving the sperm count, probably through improved spermatogenesis or facilitated sperm maturation (Khaw et al., 2020).

Another cause of poor sperm quality is the fragmentation of the DNA of spermatozoa (Agarwal et al., 2016), which is usually tracked back to a defective meiosis during the first steps of spermatogenesis. Retinoic acid is the active form of retinol, and it is essential during spermatogenesis to produce mature spermatozoa from undifferentiated germ cells due to its role as a meiosis inducer (Gewiss et al., 2021; Hogarth & Griswold, 2010, 2013). When comparing the retinol/retinoic acid presence in both groups of the present study we found that, quite surprisingly, the high sperm count group showed the lowest levels of retinoic acid. This counterintuitive finding shall be considered in a spatial context. Even if higher levels of retinoic acid must be present in the seminiferous tubes to promote sperm cell development, it does not mean that the same retinol-to-retinoic acid ratio should be preserved in the seminal plasma. Indeed, the presence and use of the active form by developing sperms could turn the balance towards retinol when it comes to the amount of each molecule being able to make their way from the seminiferous tubes to the final composition of seminal liquid.

Homovanillinic acid is a degradation metabolite from dopamine. It has been found that intracellular accumulation of dopamine in sperm cells reduces their mobility, maybe through the production of oxidative species (Ramírez-Reveco et al., 2017), and homovanillinic acid itself has been previously found to be less concentrated in patients showing fertility issues (Chen et al., 2015). Earlier literature has shown that increased itaconate production in oxidative phosphorylation regulates the transition from glycolysis to pentose phosphate pathway transition to maintain redox homeostasis, playing a role in improving a high mobility rate in these cells (Zhu et al., 2020). Although changes observed in the present study mainly relate to sperm count, they point towards the contribution of these metabolites in spermatogenesis and sperm energy production.

## Conclusions

In the present study, a subset of 210 seminal fluid samples from a nation-wide study on semen quality has been characterized by using an untargeted metabolomics approach. The detection of steroids and other low-to-mid-polarity compounds allowed the identification of metabolic differences between men having low and high total sperm counts. Other parameters such as the sperm morphology did not show clear relationship with the metabolomic profile. An uninformative variable removal strategy based on iterative Monte Carlo subsampling allowed to boil down the initial panel of annotated metabolites to the most relevant 87 ones. Some of the highlighted molecules are known to play a role in sexual development, inflammatory signaling, and sperm cell maturation and preservation, thus showing the potential of untargeted metabolomics to get a deeper insight into the mechanisms underlying cell count decrease and, in general, male fertility impairment. Opening metabolomics analyses to other groups of compounds such as polar metabolites will improve their capacity to provide an insight into this condition.

## Supplementary Information

Below is the link to the electronic supplementary material.

Supplementary file1 (XLSX 24 KB)

Supplementary file2 (XLSX 27 KB)

## Data Availability

Data can be obtained from the authors upon request.
